# Herbicides Tolerance in a *Pseudomonas* Strain Is Associated With Metabolic Plasticity of Antioxidative Enzymes Regardless of Selection

**DOI:** 10.3389/fmicb.2021.673211

**Published:** 2021-06-22

**Authors:** Amanda Flávia da Silva Rovida, Gessica Costa, Mariana Inglês Santos, Caroline Rosa Silva, Paloma Nathane Nunes Freitas, Elizangela Paz Oliveira, Sônia Alvim Veiga Pileggi, Ricardo Luiz Olchanheski, Marcos Pileggi

**Affiliations:** ^1^Department of Biotechnology, Genetics and Cell Biology, State University of Maringá, Maringá, Brazil; ^2^Laboratory of Environmental Microbiology, Biological and Health Sciences Sector, Department of Structural and Molecular Biology and Genetics, State University of Ponta Grossa, Ponta Grossa, Brazil

**Keywords:** antioxidant enzymes, oxidative stress, selective pressure, bacterial adaptation, herbicide bioremediation, herbicide degradation, metabolic plasticity

## Abstract

Agriculture uses many food production chains, and herbicides participate in this process by eliminating weeds through different biochemical strategies. However, herbicides can affect non-target organisms such as bacteria, which can suffer damage if there is no efficient control of reactive oxygen species. It is not clear, according to the literature, whether the efficiency of this control needs to be selected by the presence of xenobiotics. Thus, the *Pseudomonas* sp. CMA 6.9 strain, collected from biofilms in an herbicide packaging washing tank, was selected for its tolerance to pesticides and analyzed for activities of different antioxidative enzymes against the herbicides Boral^®^, absent at the isolation site, and Heat^®^, present at the site; both herbicides have the same mode of action, the inhibition of the enzyme protoporphyrinogen oxidase. The strain showed tolerance to both herbicides in doses up to 45 times than those applied in agriculture. The toxicity of these herbicides, which is greater for Boral^®^, was assessed by means of oxidative stress indicators, growth kinetics, viability, and amounts of peroxide and malondialdehyde. However, the studied strain showed two characteristic antioxidant response systems for each herbicide: glutathione-s-transferase acting to control malondialdehyde in treatments with Boral^®^; and catalase, ascorbate peroxidase, and guaiacol peroxidase in the control of peroxide induced by Heat^®^. It is possible that this modulation of the activity of different enzymes independent of previous selection characterizes a system of metabolic plasticity that may be more general in the adaptation of microorganisms in soil and water environments subjected to chemical contaminants. This is relevant to the impact of pesticides on the diversity and abundance of microbial species as well as a promising line of metabolic studies in microbial consortia for use in bioremediation.

## Introduction

The herbicides used to combat weeds have become indispensable in agriculture, increasing productivity but also causing several environmental problems, such as impacts on non-target organisms (Thiour-Mauprivez et al., 2019).

Bacterial communities can undergo intense changes in their diversity when in contact with herbicides, which can affect soil and water quality, as in cases of exposure to 2,4-D (Moretto et al., 2017; Meena et al., 2020), atrazine, diuron (Moretto et al., 2017) and mesotrione (Du et al., 2018), by triazines in groundwater (Mauffret et al., 2017), and atrazine, glyphosate, malathion, carbaryl, and permethrin in container aquatic habitats (Muturi et al., 2017). The herbicide alachlor caused a reduction in biomass and inhibited bacterial growth in river waters collected from the Saskatchewan River, Saskatoon, SK, Canada, when they were inoculated in bioreactors (Paule et al., 2016).

When subjected to stressful environmental conditions, bacterial strains can naturally produce reactive oxygen species (ROS) in large quantities. Contaminants, such as herbicides, cause oxidative stress through the generation of ROS, which interact with the cell membrane and can cause lipid peroxidation (Dourado et al., 2015). As a way of preventing or reducing these types of imbalances, bacteria have developed response systems to protect membrane integrity, such as modulating the activity of antioxidant enzymes (Martins et al., 2011; Lemire et al., 2017). Bacteria can become tolerant to oxidative stress by secreting extracellular polymeric substances (EPS) and forming biofilms (Tolker-Nielsen, 2015). The efficiency of these responses can lead to different levels of susceptibility and sensitivity of bacterial communities to stressors, leading to changes in their diversity and ecological functions (Polst et al., 2018).

Microorganisms have stress response mechanisms, including increased expression of enzymes that transform ROS, such as superoxide dismutase (SOD), which catalyzes the dismutation of superoxide (O_2_^.–^) into oxygen (O_2_) and hydrogen peroxide (H_2_O_2_), and catalase (CAT), which decomposes H_2_O_2_ into water (H_2_O) and oxygen (O_2_) (Prione et al., 2016), in addition to peroxidases such as glutathione S-transferase (GST) (Wongsaroj et al., 2018). However, there are no in-depth studies analyzing the efficiency of this mechanism in the presence of xenobiotics. Even for some enzymes, there are no recent reports of the effects on bacteria, but only indirect on enzymes from bacteria-colonized plants, such as guaiacol peroxidase (GPX) (Eke et al., 2019), and ascorbate peroxidase (APX) (Fan et al., 2020).

The genus *Pseudomonas* stands out for its tolerance to chemical stressors because of its metabolic and physiological versatility, and it can be isolated from soil, fresh water, biofilms, and other places, with potential for application in bioremediation (Venturi, 2006; Karami et al., 2016; Melo et al., 2016). Lima et al. (2020) obtained collections of bacteria capable of tolerating different herbicides, including *Pseudomonas* sp. CMA 6.9, assessed in this study. This strain was isolated from a tank containing water for washing pesticide packaging in which the Heat^®^ herbicide was present.

Boral^®^ and Heat^®^ are herbicides whose mode of action inhibits the enzyme protoporphyrinogen oxidase (protox) (Beffa et al., 2019). These herbicides have electronegative and oxidizing chemical elements, such as fluorine (F) and chlorine (Cl), that can affect the stress response genes in microorganisms (Li et al., 2020), compromise the physical structure, and affect metabolic processes, efflux pumps, and regulation of gene expression (Ye et al., 2020).

The use of cycles of chemical substances, such as fungicides, insecticides, antibiotics, and herbicides, is considered key for the selection of resistance in organisms through specific strategies, based on the mode of action of these substances. These are the target-site resistance (TSR) models. New proposals have been designed, called non-target-site resistance (NTSR), and are based on related genes in different systems of detoxification, transport, efflux, and sequestration. These systems can give resistance to chemical substances with different modes of action, being, therefore, more general mechanisms (Comont et al., 2020). Metabolic mechanisms, described in plants are also included in NTSR, such as those reactions involving the esterase enzymes, GSTs, uridine 5′ diphospho-glucosyl transferases, and cytochrome P450s (Jugulam and Shyam, 2019; Gaines et al., 2020).

This study aimed, therefore, to compare the mode of modulation of antioxidant mechanisms of *Pseudomonas* sp. CMA 6.9 in the presence of the herbicide Boral^®^, which was absent at the isolation site of this strain, and of the Heat^®^, which exerts selective pressure at the isolation site of this strain.

## Materials and Methods

**FIGURE 1:**
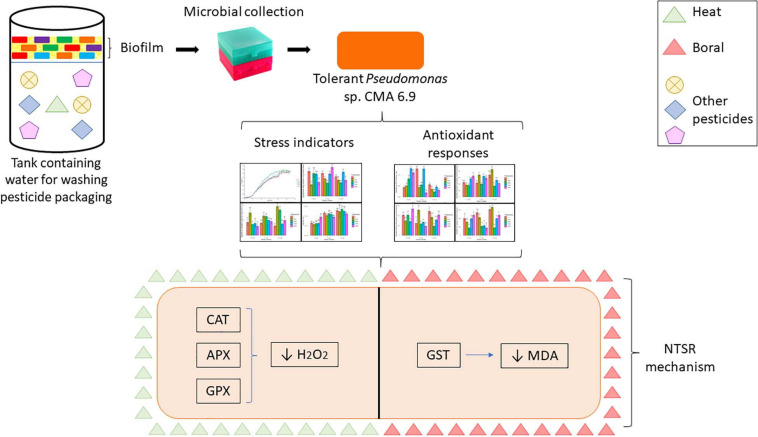
Experimental design showing the tests performed for the preliminary characterization of the lineage and evaluations of oxidative stress indicators and stress response systems.

In accordance with the objectives of this work, the following experimental design (Figure 1) was carried out.

### Bacterial Strain

*Pseudomonas* sp. CMA 6.9 was from the Collection of Environmental Microorganisms of the Laboratory of Environmental Microbiology of the State University of Ponta Grossa (UEPG). This collection consists of 67 bacterial biofilm isolates from water storage tanks used to wash agrochemical packaging (Lima et al., 2020) at the BASF experimental station, Fazenda Escola Capão da Onça of UEPG.

### Herbicides

The commercially sold herbicides Boral^®^ and Heat^®^ were used in this work.

The Boral^®^ 500 SC herbicide (FMC, Philadelphia, PA, United States) contains 500 g/L (50% w/v) of the active ingredient sulfentrazone (2′,4′-dichloro-5′ - (4-difluoromethyl-4,5-dihydro-3-methyl-5-oxo-1H-1,2,4-triazole-1-yl) methanesulfonanilide). The doses used in the experiments were 15x (B15) and 45x (B45) the concentration used in the field, with concentrations of 7.3 mMol and 22.0 mMol, respectively.

The herbicide Heat^®^ (BASF, Ludwigshafen, Rhein, Germany) contains 700 g/L (70% w/v) of the active ingredient saflufenacil (N′- {2-chloro-4-fluoro-5- [1,2,3, 6-tetrahydro-3-methyl-2,6-dioxo-4- (trifluoromethyl) pyrimidin-1-yl] benzoyl} -N-isopropyl-N-methylsulfamide). The doses used in the experiments were 15x (H15) and 45x (H45) at concentrations of 0.7 mMol and 2.2 mMol, respectively.

### Herbicide Tolerance Test

The herbicides were added to Luria Bertani Agar (LA) at 45°C. The treatments H15, H45, B15, and B45 were compared to the control (C) in triplicate without herbicides. The plates were incubated at 30°C for 24 h. Isolates that grew in culture medium were considered tolerant.

### Molecular Identification by Sequencing the 16S Ribosomal Gene

The strain was grown in a plate containing LA. After incubation for 48 h at 30°C, isolated colonies were collected for molecular identification. The identity of the isolate was determined by sequencing the 16S ribosomal gene. The total DNA was extracted using the DNA isolation kit from Promega (Madison, WI, United States). Primers fD1 (5′-CCGAATTCGT CGACAACAGAGTTTGATCCTGGCTCAG-3′) and rD1 (5′-CCCGGGATCCAAGCTTAAGGAGGTGATCCAGCC-3′) were used for amplification (Benson et al., 1996). This reaction consisted of an initial denaturation cycle at 95°C for 5 min, 94°C for 45 s, annealing at 55°C for 45 s, extension at 72°C for 2 min, and a final extension cycle for 10 min at 72°C. The PCR products were purified using the QIAquick PCR kit (no. 28104). After purifying the PCR products and analyzing the integrity of the bands using electrophoresis, the material obtained was sent for sequencing at Ludwig Biotec (Alvorada, Brazil). The sequences were analyzed using the resources of the “Ribosomal Database Project” site. The strain was identified as belonging to the genus *Pseudomonas*, accession number MT072022 – NCBI, named as *Pseudomonas* sp. CMA 6.9. It is deposited at the Center for Biological Resources Johanna Döbereiner (CRB-JD) – Embrapa Agrobiologia, under the code BR 14567 and is part of the UEPG’s Collection of Environmental Microorganisms.

### Oxidative Stress Analysis

#### Bacterial Growth Curve

The bacterial strain was incubated at 30°C at 120 rpm overnight in Luria Bertani Broth (LB) to produce the pre-inoculum. Thereafter, the inoculum was placed in 250-ml flasks containing 100 ml of the treatments in LB at an initial optical density (OD) of 0.05–600 nm. Bacterial growth was monitored every 1 h for 27 h in a spectrophotometer at 600 nm. The growth values were obtained multiplying the OD obtained in a diluted suspension of bacteria by the dilution factor. The experiments were conducted in triplicate.

#### Cell Viability

After preparing the pre-inoculum (Section “Bacterial Growth Curve”), the inoculum was transferred to the control and treatments B15, B45, H15, and H45. Aliquots of 100 μL were removed in three growth phases established from the growth curves (6 h, 9 h, and 12 h). The samples were diluted sequentially in the proportion of 1–10, inoculated on LA, and incubated at 30°C. After 24 h, colony forming units (CFU) were counted, containing 25–300 colonies.

#### Sample Preparation for Cell Damage

After preparing the pre-inoculum (Section “Bacterial Growth Curve”), the cells were cultured in the established phases (Section “Cell Viability”). The cultures were centrifuged at 8000 × *g* for 10 min at 4°C, the supernatant was discarded, and the precipitate was macerated in liquid nitrogen. Then 100-mg aliquots of samples were frozen at −80°C until analyzed (Alexieva et al., 2001).

##### Quantification of H_2_O_2_

The extracted samples were homogenized with 1 ml of trichloroacetic acid (TCA) to 0.1% and centrifuged at 10,000 × *g* for 15 min at 4°C. Subsequently, 0.2 mL of the supernatant was collected and transferred to a tube containing 0.2 mL of a 100 mM solution of potassium phosphate buffer (14.52 g/L K_2_HPO_4_, 2.26 g/L KH_2_PO_4_, pH 7.5) and 0.8 mL of 1 M potassium iodide (KI) solution. The sample was stored in the dark and on ice for 1 h. The reading was performed on a spectrophotometer at 390 nm. The results were expressed in μmol H_2_O_2_/g of fresh weight (Dourado et al., 2013).

##### Quantification of lipid peroxidation

Quantification of lipid peroxidation was carried out by measuring the release of malondialdehyde (MDA), a reactive metabolite of 2-thiobarbituric acid (TBA) (Heath and Packer, 1968, with modifications). For this test, 0.1 g of each sample (Section “Quantification of H_2_O_2_”) was homogenized with 1 ml of TCA to 0.1% and centrifuged at 11,600 × *g* for 5 min. The samples were collected in a 0.25 mL aliquot of supernatant and transferred to a test tube containing 1 ml of 20% TCA and 0.5% TBA. The reaction mixture was placed in a dry bath for 30 min at 95°C, cooled on ice for 10 min, and centrifuged at 11,600 × *g* for 10 min. The MDA concentration was monitored at 535 nm (all lipids) and 600 nm (all lipids except for MDA) and calculated using an extinction coefficient of 155 mM/cm. The amount of MDA was expressed in μmol MDA/g fresh weight.

#### Protein Extraction for Oxidative Stress Analysis

After pre-inoculation preparation (Section “Bacterial Growth Curve”), cultures were centrifuged at 8,000 × *g* for 10 min. The precipitate was macerated in liquid nitrogen, homogenized (10:1 w/v) in 100 mM potassium phosphate buffer (pH 7.5) containing 1 mM ethylene diamine tetra-acetic acid (EDTA), 3 mM dithiothreitol (DTT), and 5% (w/v) polyvinylpolypyrrolidone and kept at 4°C. The homogenate was centrifuged at 10,000 × *g* for 30 min, and the supernatant was collected and frozen for further enzymatic analysis. The protein concentration was determined using bovine serum albumin (BSA) as a standard (Bradford, 1976). The results were expressed as μmol of protein/g of fresh weight.

##### Total proteins – SDS

As a quality control of the control protein extracts and treatments (Section “Protein Extraction for Oxidative Stress Analysis”), SDS-polyacrylamide gel electrophoresis (PAGE) gel (Supplementary Material 1) was performed. The electrophoresis experiments in discontinuous and denaturing buffer systems were performed using an 8.3 × 10.2 cm mini-gel system at 10% acrylamide concentration (Laemmli, 1970). Protein denaturation was performed by heating at 95°C for 5 min, and 10% SDS was used in the running buffer and in the packaging buffer. For resolution, the gels were washed in distilled water and incubated overnight in 0.05% Coomassie blue R-250 in water/methanol/acetic acid 45/45/10 (v/v/v) and color removed by successive washing in the water/methanol/acetic acid 45/45/10 (v/v/v) solution.

##### Determination of SOD isoforms

We performed 12% non-denaturing polyacrylamide gel electrophoresis (PAGE) with protein extract (Section “Protein Extraction for Oxidative Stress Analysis”), and the gel was divided vertically into three parts. The first part was maintained at 4°C in 100 mM potassium phosphate buffer, pH 7.8. The second was immersed in 100 ml of the same buffer containing 2 mM KCN and 0.0292 g of EDTA. The third was immersed in 100 ml of said buffer containing 5 mM H_2_O_2_ and 0.0292 g of EDTA. The experiments were carried out in the dark. After 20 min in these solutions, the gels were stained with nitroblue tetrazolium (NBT) and riboflavin, as previously mentioned, and bands appeared. Isoforms were classified as Mn-SOD if resistant to both inhibitors (KCN and H_2_O_2_), Fe-SOD if resistant to KCN and inhibited by H_2_O_2_, and Cu/Zn-SOD if inhibited by both substances (Azevedo et al., 1998).

##### PAGE SOD activity

Electrophoresis was performed on non-denaturing polyacrylamide separating gels at 12% and a stacking gel of polyacrylamide at 4% at a current of 15 mA for 3 h. A 20-ng aliquot of protein (Section “Protein Extraction for Oxidative Stress Analysis”) was applied to each well. SOD activity was determined as described by Peters et al. (2014). The gels were washed in distilled water and incubated in the dark for 30 min in 50 mM potassium phosphate buffer (pH 7.8) containing 1 mM EDTA, 0.005 mM riboflavin, 0.1 mM nitroblue tetrazolium and N, N, N′, 0.03 mM N′-tetramethylethylenediamine (TEMED). The gels were exposed to white light and immersed in water until the bands corresponding to the SOD bands became visible. Since there are differences between measurements of enzymatic activities (Faria et al., 2015), in the case of SOD we prefer to use the electrophoretic method to the enzymatic one.

##### CAT activity

Catalase activity was determined in a solution containing 1 ml of 100 mM potassium phosphate buffer (pH 7.5) and 2.5 μL de H_2_O_2_ (30% solution) and quantified in a spectrophotometer at 25°C. The reaction was started with the addition of 50 μL of protein extract (Section “Protein Extraction for Oxidative Stress Analysis”), and the activity was determined following the decomposition of H_2_O_2_ at 240 nm for 1 min using the molar extinction coefficient of 43.6 M^–1^ cm^–1^. The results were expressed in μmol/min/mg protein (Kraus et al., 1995).

##### APX activity

Ascorbate peroxidase activity was determined in a reaction mixture with 650 μL of potassium phosphate buffer (80 mM, pH 7.0), 100 μL EDTA (1 mM), 100 μL ascorbic acid (5 mM), 100 μL H_2_O_2_ (1 mM) and quantified in a spectrophotometer at 25°C. The reaction was started with the addition of 50 μL of protein extract (Section “Protein Extraction for Oxidative Stress Analysis”) and the activity was determined following the decomposition of H_2_O_2_ by APX at 290 nm for 1 min assuming a molar extinction coefficient of 2.8 mM^–1^ cm^–1^. The activity was expressed in μmol/min/mg protein (Nakano and Asada, 1981).

##### GPX activity

Guaiacol peroxidase activity was determined by adding 25 μL of protein extract (section “Protein Extraction for Oxidative Stress Analysis”) to a solution of 390 μL of citrate buffer (0.2 M of dibasic disodium phosphate and 0.1 M of citric acid, pH 5.0), 25 μL of guaiacol, and 25 μL of H_2_O_2_ (3%), which were homogenized and immersed in a water bath at 30°C for 15 min. Then 25 μL of sodium metabisulfite (2%) was added and GPX activity was measured in a spectrophotometer at 450 nm. The activity was expressed in μmol/min/mg protein (Matsuno and Uritani, 1972).

##### GST activity

Glutathione S-transferase activity was measured in a solution containing 900 μL of 100 mM potassium phosphate buffer (pH 6.8), adding 25 μL of 40 mM 1-chloro-2,4-dinitrobenzene (CDNB) and 50 μL of reduced glutathione (GSH) 0.1 M and incubating at 30°C (Zablotowicz et al., 1995). The reaction was started with the addition of 25 μL of protein extract (Section “Protein Extraction for Oxidative Stress Analysis”) and was monitored for 2 min at 340 nm. The activity was expressed in μmol/min/mg protein.

### Statistical Analysis

Data on quantification of the growth curve, H_2_O_2_, MDA, CAT, APX, and GPX were obtained in triplicate for each treatment and statistically analyzed using a randomized complete block design. Significance analyses were obtained with the R 1.3.1056 program using the Duncan test (*p* < 0.05) (Supplementary Material 2). All these data were processed by principal component analysis (PCA) (Gotelli et al., 2011).

## Results

### Herbicide Tolerance Test

The evaluation of tolerance to the Boral^®^ and Heat^®^ herbicides showed that of the 67 isolates tested, 45 (67.2%) did not show growth and therefore were considered sensitive to these herbicides. The 22 (32.8%) that were herbicide tolerant were grown on LB + herbicides and based on OD data, the most tolerant strain was identified as *Pseudomonas* sp. CMA 6.9.

### Indicators of Oxidative Stress in Bacteria

#### Data on Growth Kinetics of *Pseudomonas* sp. CMA 6.9 in Media Containing Herbicides

**FIGURE 2:**
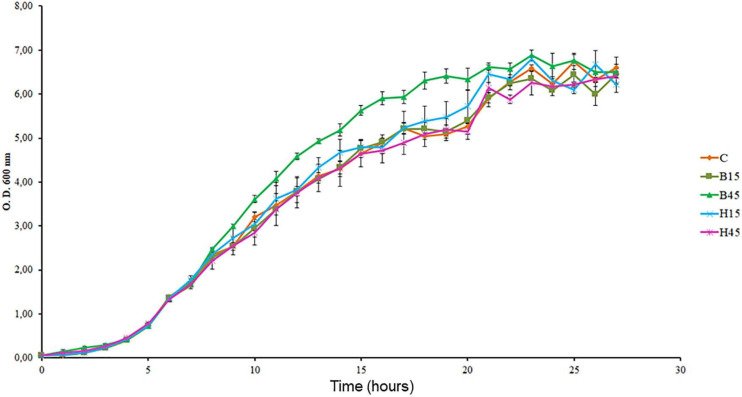
Growth curves of *Pseudomonas* sp. CMA 6.9 in control treatments (C) and with the herbicides Boral^®^ (B15 and B45) and Heat^®^ (H15 and H45), determined by OD at 600 nm. Data were obtained in triplicate for each treatment and statistically analyzed using a randomized complete block design using the Duncan test (*p* < 0.05). Error bars represent standard errors of the means.

The growth rates of *Pseudomonas* sp. CMA 6.9 in the treatments were significantly greater or equal in comparison to the control (Figure 2 and Supplementary Material 2). Based on the growth curves, three phases were established in the logarithmic period in which the samples were obtained, and analyses were made of stress indicators and enzyme response systems as follows: early-log (at 6 h of incubation), early mid-log (at 9 h), and mid-log (in 12 h).

#### Viability Data for *Pseudomonas* sp. CMA 6.9 Against Boral^®^ and Heat^®^ Herbicides

**FIGURE 3:**
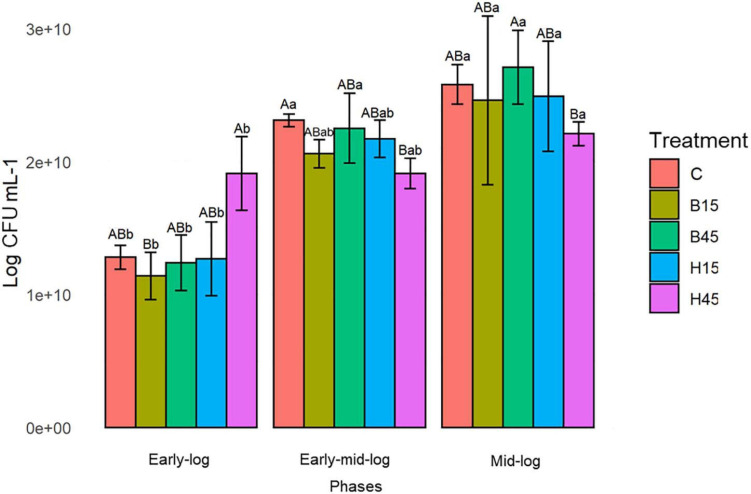
Cell viability of *Pseudomonas* sp. CMA 6.9 in control treatments (C) and with the herbicides Boral^®^ (B15 and B45) and Heat^®^ (H15 and H45) in the three growth phases indicated. Data were obtained in triplicate for each treatment and statistically analyzed using a randomized complete block design using the Duncan test (*p* < 0.05). Error bars represent standard errors of the means. Different uppercase letters represent statistically significant differences between treatments at the same time; lower-case letters represent statistically significant differences between treatments at different times.

Viability data were measured in *Pseudomonas* sp. CMA 6.9 under the described growth conditions (Section “Bacterial Growth Curve”). The feasibility data (Figure 3) showed significant increases over the three evaluated periods (Supplementary Material 2).

#### Quantification of H_2_O_2_

**FIGURE 4:**
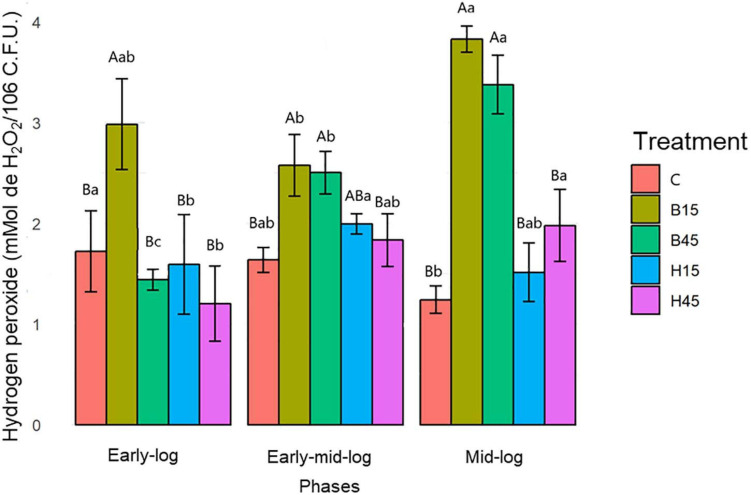
Quantification of H_2_O_2_ from *Pseudomonas* sp. CMA 6.9 in control treatments (C) and with the herbicides Boral^®^ (B15 and B45) and Heat^®^ (H15 and H45) in the three growth phases indicated. Data were obtained in triplicate for each treatment and statistically analyzed using a randomized complete block design using the Duncan test (*p* < 0.05). Error bars represent standard errors of the means. Different uppercase letters represent statistically significant differences between treatments at the same time; lower-case letters represent statistically significant differences between treatments at different times.

The concentrations of H_2_O_2_ found to s in *Pseudomonas* sp. 6.9 CMA were significantly lower in control than in treatments with Boral^®^ and Heat^®^ most prevalently in the early stage. The concentrations of H_2_O_2_ in the treatments with Boral^®^, especially in the mid-log stage of growth, were significantly greater (Supplementary Material 2) than in the other treatments (Figure 4).

#### Quantification of MDA

**FIGURE 5:**
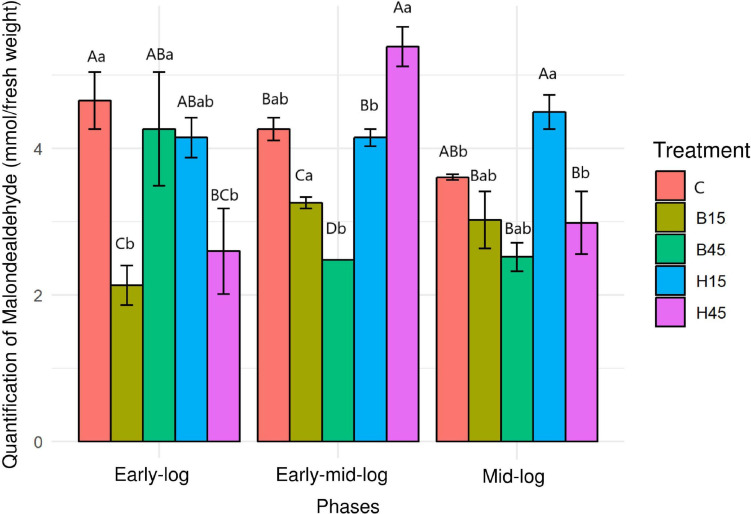
Quantification of MDA from *Pseudomonas* sp. CMA 6.9 in control treatments (C) and with the herbicides Boral^®^ (B15 and B45) and Heat^®^ (H15 and H45) in the three growth phases indicated. Data were obtained in triplicate for each treatment and statistically analyzed using a randomized complete block design using the Duncan test (*p* < 0.05). Error bars represent standard errors of the means. Different uppercase letters represent statistically significant differences between treatments at the same time; lower-case letters represent statistically significant differences between treatments at different times.

The concentration of MDA in the controls and treatments with Boral^®^ and Heat^®^ was evaluated as an indicator of lipid peroxidation and cytotoxicity caused by these herbicides (Figure 5).

The concentrations of MDA in the control showed significant decreases over the analyzed growth periods. In general, the concentrations in treatments with Heat^®^ were significantly higher, mainly in the early mid-log phase in the H45 treatment, while concentrations in the treatments with Boral^®^ were significantly lower in relation to the control.

### Activities of Antioxidative Enzymes in *Pseudomonas* sp. CMA 6.9

The *Pseudomonas* sp. CMA 6.9 in response to the toxicity of Boral^®^ and Heat^®^ herbicides was evaluated by quantifying the activities of the enzymes SOD, CAT, APX, GPX, and GST.

#### SOD Enzymatic Activity

**FIGURE 6:**
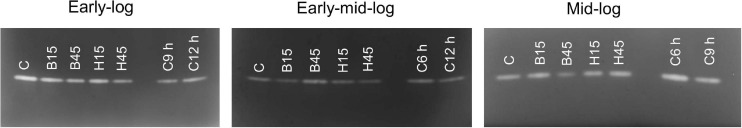
SOD activity of *Pseudomonas* sp. CMA 6.9 in control treatments (C) and with the herbicides Boral^®^ (B15 and B45) and Heat^®^ (H15 and H45) in the three growth phases indicated. In each of the growth phases, replicates of the other controls were made, with C6 h, 6 h control; C9 h, 9 h control; C12 h, 12 h control.

Iron superoxide dismutase (Fe-SOD) was the only isoform characterized for *Pseudomonas* sp. CMA 6.9 (data not shown). There were no significant variations in activity between control and treatments, except for B45, which showed a greater variation in the relative band intensity in the three growth phases evaluated (Figure 6).

#### CAT, APX and GPX Enzymatic Activities

**FIGURE 7:**
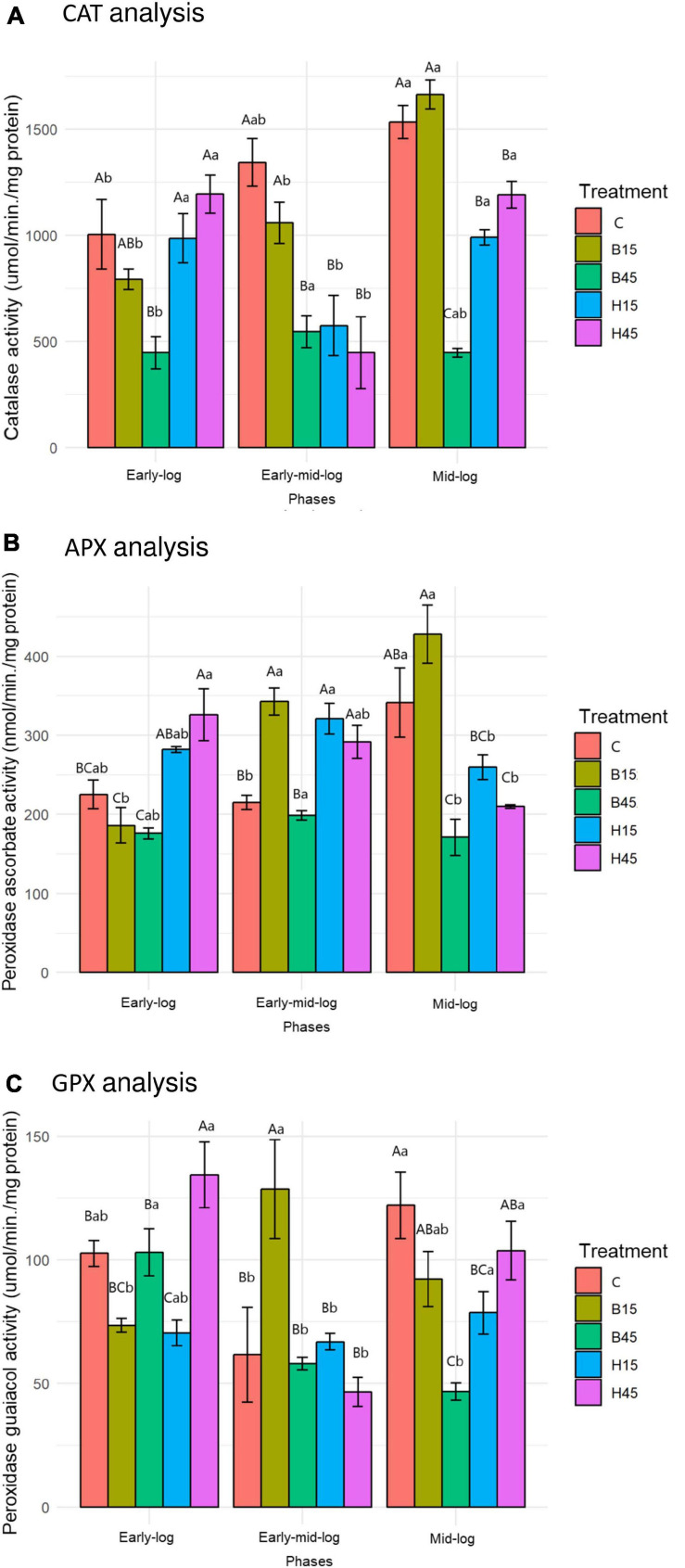
CAT **(A)**, APX **(B)**, and GPX **(C)** activity of *Pseudomonas* sp. CMA 6.9 in control treatments (C) and with the herbicides Boral^®^ (B15 and B45) and Heat^®^ (H15 and H45) in the three growth phases indicated. Data were obtained in triplicate for each treatment and statistically analyzed using a randomized complete block design using the Duncan test (*p* < 0.05). Error bars represent standard errors of the means. Different uppercase letters represent statistically significant differences between treatments at the same time; lower-case letters represent statistically significant differences between treatments at different times. CAT, catalase; APX, ascorbate peroxidase; GPX, guaiacol peroxidase.

The activities of CAT, APX, and GPX were analyzed in this section because they are enzymes with the H_2_O_2_ metabolizing function; the data are shown in Figure 7.

The enzymes CAT, APX, and GPX showed a significant increase in activities throughout the growth phases, analyzing the behavior in control. The treatments with the Boral^®^ herbicide were also characterized by higher activities of the three enzymes, mainly in B15, and in general increasing throughout the growth phases. In the B45 treatment, the enzyme activities were generally lower than in the control and B15. Variations in the activities of the three enzymes in the studied growth phases were similar. In treatments with the herbicide Heat^®^, however, the general behavior of enzymatic activities was to decrease significantly.

#### Enzymatic Activity of GST

**FIGURE 8:**
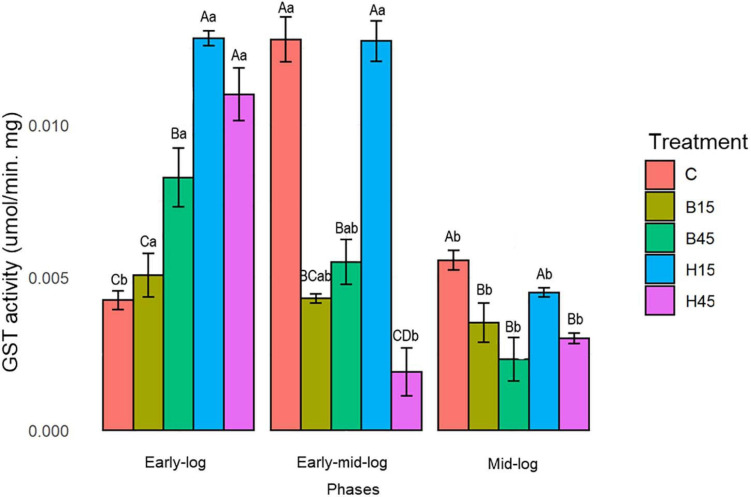
GST activity of *Pseudomonas* sp. CMA 6.9 in control treatments (C) and with the herbicides Boral^®^ (B15 and B45) and Heat^®^ (H15 and H45) in the three growth phases indicated. Data were obtained in triplicate for each treatment and statistically analyzed using a randomized complete block design using the Duncan test (*p* < 0.05). Error bars represent standard errors of the means. Different uppercase letters represent statistically significant differences between treatments at the same time; lower-case letters represent statistically significant differences between treatments at different times.

Quantitative analysis of GST for *Pseudomonas* sp. CMA 6.9 is shown in Figure 8.

Glutathione S-transferase activity for *Pseudomonas* sp. CMA 6.9, under control conditions, showed significantly higher activity in the early mid-log phase compared to other growth phases. In treatments with the Boral^®^ herbicide, there was a drop-in activity throughout the growth phases, with a more pronounced drop for B45. With the Heat^®^ treatment, the variation in enzyme activity was more complex, depending on the concentration of the herbicide. For H15, the high activities, which were higher among the different experiments, followed a significant drop in the mid-log phase. In H45, the high rate in the early phase was followed by a significant drop in the early mid-log and mid-log phase.

### Interrelationships Between Stress Indicators and Enzymatic Activities in *Pseudomonas* sp. CMA 6.9 in Treatments With Boral^®^ and Heat^®^

**FIGURE 9:**
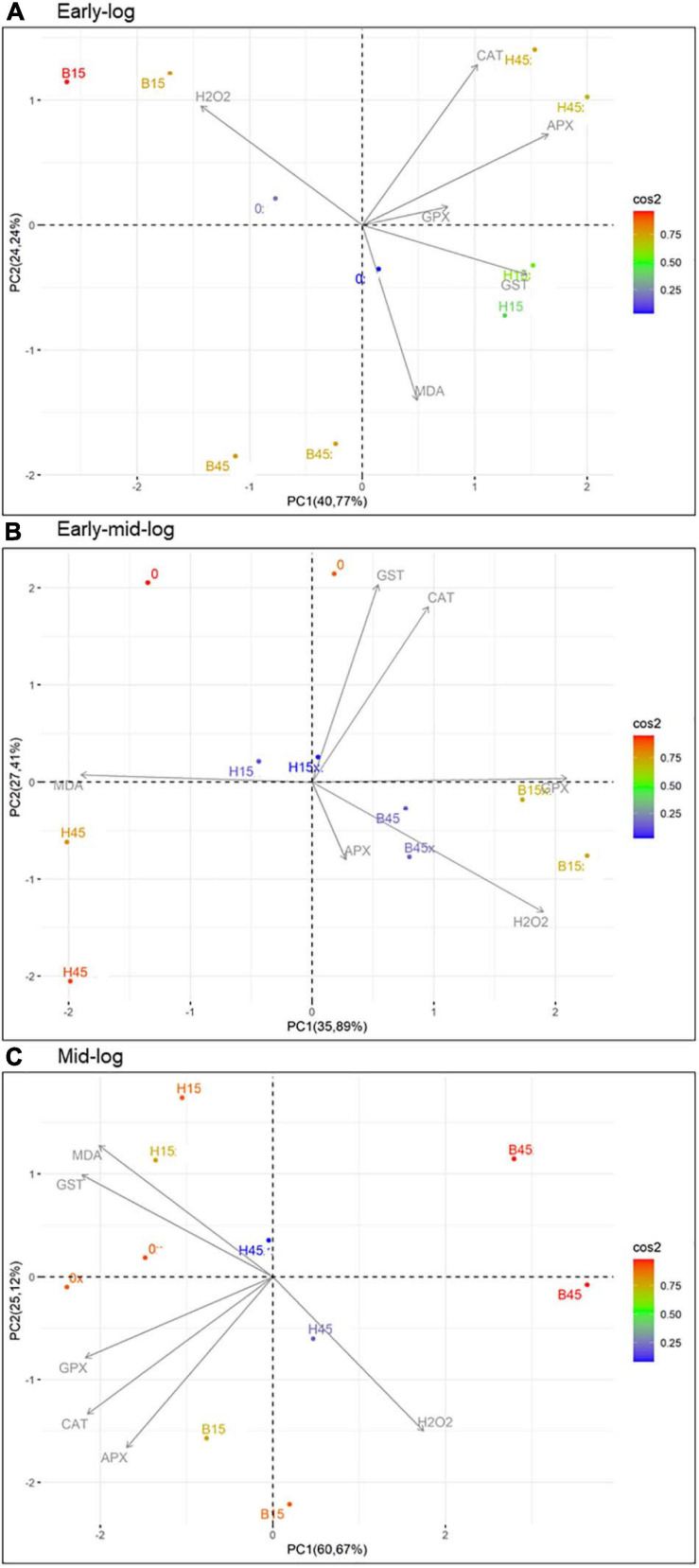
PCA of the relationships between quantifications of stress indicators H_2_O_2_ and MDA and profiles of enzymatic activities of CAT, APX, GPX, and GST of *Pseudomonas* sp. CMA 6.9 in the three growth phases indicated. **(A)** Early-log phase; **(B)** Early mid-log phase; **(C)** Mid-log. Color scale indicates the degree of contribution of the data distribution. CAT, catalase; APX, ascorbate peroxidase; GPX, guaiacol peroxidase; GST, glutathione S-transferase.

Principal component analysis was used to assess data interrelationships in the quantification of stress indicators, H_2_O_2_ and MDA, and activities of the antioxidant enzymes CAT, APX, GPX, and GST (Figure 9). The most significant interrelationships were important for the elaboration of the enzymatic response systems of *Pseudomonas* sp. CMA 6.9 against the toxicity of herbicides Boral^®^ (not present in the isolation tanks) and Heat^®^ (present in the isolation tanks).

## Discussion

### Tolerance to Herbicides by *Pseudomonas* sp. CMA 6.9

The tolerance evaluation of bacteria obtained in tanks containing washing water from pesticide containers (Lima et al., 2020) showed that 67.2% of the isolates were sensitive to herbicides Boral^®^ and Heat^®^ at 15x and 45x concentrations. It is known that herbicides can be toxic for non-target organisms because of the electronegative characteristics of their active molecules (Pileggi et al., 2020) and can reduce microbial diversity, such as that of *Actinobacter* in the presence of Granstar, a sulfonylurea herbicide, and that prior exposure to the herbicide acts as a selective agent (Rachedi et al., 2018). However, the 32.8% of tolerant isolates to these herbicides, from which the *Pseudomonas* sp. 6.9 CMA was selected, suggests that there are adaptation mechanisms that do not need specific selective agents as inducers. No articles were found on bacterial strains carrying tolerance systems without prior selection events. Sensitive bacteria can be protected in communities structured in biofilms, where a part of the community can contain tolerance systems, but selected by stressors, such as the case of marine periphytic biofilms under ecotoxicological effect of copper (Corcoll et al., 2019).

### Indicators of Oxidative Stress in Bacteria

Oxidative stress occurs when there is an imbalance between the production of ROS and their removal by enzymatic and non-enzymatic action, by which more toxic molecules are transformed into less harmful ones (Imlay, 2019). Population growth kinetics, cell viability, and quantification of H_2_O_2_ and MDA were evaluated as indicators of oxidative stress induced by herbicides, since it is important to let it be characterized that these substances, even when targeting weeds, are toxic to bacteria.

#### Growth Kinetics

The times corresponding to the first phases of logarithmic growth were determined to evaluate the differences in the induction of the response systems of the strain to the two herbicides, because the growth phases indicate the adaptability of the strain to the medium (Molina et al., 2019; Ram et al., 2019). The early-log (6 h), early mid-log (9 h), and mid-log (12 h) phases were standardized to obtain data on stress indicators and enzyme responses. According to the data obtained, such as growth rates of *Pseudomonas* sp. CMA 6.9 in control was not significantly higher than in the treatments with herbicides, this strain was considered tolerant to Boral^®^ and Heat^®^, and the isolates that did not show any growth, even in the presence of 1x the herbicides, were considered sensitive.

#### Viability of *Pseudomonas* sp. CMA 6.9

Cell viability is one of the most important indicators for determining whether cells are under oxidative stress and undergoing damage (Ye et al., 2019). Stressors such as herbicides can decrease cell viability. The cyanobacterium *Microcystis aeruginosa*, for example, when exposed to glyphosate showed an increase in oxidative stress levels, indicated by MDA and cell apoptosis, and consequently, a decrease in cell viability levels, even with an increase in the activity of antioxidant enzymes such as SOD, CAT, and peroxidases (Wu et al., 2016). The collection that was screened in this work showed 67.2% of its isolates were herbicide-sensitives. Even so, no data were found in the literature on the effects on Boral^®^ and Heat^®^ on the viability of bacteria. In contrast, *Pseudomonas* sp. CMA 6.9 showed significant increases in growth rates (Figure 2) and viability (Figure 3) during the three time periods evaluated, indicating that it has efficient response systems for adaptation to herbicide absent at the isolation site (Boral^®^), as well as herbicide present (Heat^®^), not seen in other isolates from the same environment.

#### Quantification of H_2_O_2_

Reactive oxygen species, such as H_2_O_2_ and O_2_^–^, are generated via normal aerobic metabolic processes in the electron transport chain; however, these molecules are toxic to cells, especially when their levels are high because of the presence of stressors (Prione et al., 2016).

Boral^®^ was considered to have a greater potential to cause oxidative stress in *Pseudomonas* sp. CMA 6.9 than Heat^®^, considering the comparisons between the concentrations of H_2_O_2_ in the treatments of these herbicides and in the control. Other herbicides have this characteristic of increasing the amount of H_2_O_2_ in two strains of *Bacillus megaterium*, leading to the combined toxic effects of adjuvants and the active molecule mesotrione (Dobrzanski et al., 2018). In the same way, the herbicide gramoxone was seen to cause this effect in strains of *Escherichia coli* (Gravina et al., 2017).

#### Quantification of MDA

Malondialdehyde is an indicator of damage to unsaturated fatty acids in the membrane caused by H_2_O_2_ (Heath and Packer, 1968) and so is used as a representative biomarker of oxidative damage in ecotoxicology (Liu et al., 2019). Therefore, the concentration of MDA in the controls and treatments with Boral^®^ and Heat^®^ was evaluated as an indicator of lipid peroxidation and cytotoxicity caused by these herbicides (Figure 5). Using only these data and definitions, it would be possible to infer that Heat^®^ produces more oxidative damage to *Pseudomonas* sp. CMA 6.9 compared to Boral^®^, and this would produce less damage than in the control. Another hypothesis that could be proposed is a response related to the dose of Heat^®^ in the early mid-log phase (Figure 5), as described for *Pseudomonas stutzeri* after exposure to increasing doses of silver nanoparticles (Wu et al., 2020). However, considering the whole period of analysis and data for Boral^®^, this hypothesis cannot be confirmed.

The data on growth kinetics (Figure 2) and viability (Figure 3) show that the correlation between cytotoxicity and the amounts of MDA are also unclear. In addition, the data on the amount of H_2_O_2_ (Figure 4) and MDA (Figure 5) do not indicate a relationship between peroxide and peroxidation in the studied strain. Studies by our research group have indicated that different bacterial species can modify their lipid composition in response to the toxicity of different herbicides, changing, therefore, the proportion of unsaturated fatty acids in membranes and the amount of MDA. This has been observed for different mutated strains for SOD isoforms in *E. coli* in treatment with gramoxone (Gravina et al., 2017), for *Pantoea ananatis* (Prione et al., 2016), for *E. coli* (Olchanheski et al., 2014), and for different strains of *B. megaterium* (Dobrzanski et al., 2018), all with treatment with the herbicide mesotrione. Generally, alterations in fatty acids in the cytoplasmic membrane of Gram-negative bacteria are considered important mechanisms in adaptation to toxic compounds, such as herbicides (Murínová and Dercová, 2014).

### Enzymatic Control of Oxidative Stress Caused by the Herbicides Boral^®^ and Heat^®^ in *Pseudomonas* sp. CMA 6.9

*Pseudomonas* sp. CMA 6.9 did not degrade the active molecules of the herbicides Boral^®^ (sulfentrazone) and Heat^®^ (saflufenacil) (Supplementary Material 3); therefore, only the antioxidant enzymes were considered as a possible mechanism of response to the toxicity of these substances. Thus, enzymatic control of oxidative stress is necessary for tolerant bacteria to survive in environments in which herbicides can cause an increase in ROS and, consequently, structural and genomic damage and impaired bacteria viability. Oxidative stress is a considerable impact caused by herbicides in bacteria, such as the case of glyphosate on the intestinal microbiota of rats, according to methods of shotgun metagenomics and metabolomics, techniques with good precision to assess the presence of genes and production of related metabolites with responses to the herbicide (Mesnage et al., 2021). Therefore, to evaluate the antioxidative system of the *Pseudomonas* sp. CMA 6.9 is relevant in this context and can be considered a basis for understanding their survival as well as the sensitive isolates that cohabited biofilms in the presence of pesticides.

#### SOD Enzymatic Activity

Superoxide dismutase acts to reduce oxidative stress, catalyzing the dismutation of superoxide in O_2_ and H_2_O_2_. SOD is a metalloenzyme classified according to its metallic cofactor, such as manganese (Mn), iron (Fe), or copper-zinc (Cu-Zn). Fe-SOD was the only isoform characterized for *Pseudomonas* sp. CMA 6.9 (data not shown) for which a cytoplasmic location and constitutive synthesis is attributed (Barnes et al., 1996; Touati, 2000). The culture medium can induce differential expression of SOD isoenzymes, such as Luria Bertani, which induces the expression of Fe-SOD in *Pseudomonas aeruginosa* (Pedersen et al., 2009). There were no significant variations in activity between the control and the treatments, except for B45, which showed high activity in the early mid-log phase (Figure 9), with the probable consequence of higher production of H_2_O_2_ in the mid-log phase (Figure 4). Therefore, SOD was induced in *Pseudomonas* sp. CMA 6.9 only for Boral, but not for Heat. Regardless of the isoenzyme type, increased SOD activity in bacteria is considered an adaptive response to stress induced by some herbicides. For example, *Rhodobacter sphaeroides* and *Acinetobacter lwoffii* strains increased SOD activity in response to treatments with the herbicide atrazine (Zhang et al., 2012). This induction also occurred for a *Stenotrophomonas maltophilia* strain treated with quinclorac or bensulfuron-methyl herbicides, but not for *E. coli* (Lü et al., 2009).

#### CAT, APX and GPX Enzymatic Activities

Hydrogen peroxide has the potential to cause cell damage, thus requiring control of its concentration. The action of CAT in response to cell defense is important for bacterial adaptation and surviving herbicide-induced H_2_O_2_ (Bučková et al., 2010). CAT operates more than that produced in ROS metabolism, while APX enzymes, which use ascorbate with an electron donor, and GPX, which uses guaiacol, act to split the remaining H_2_O_2_. APX and GPX are mainly described in plants and occur in the presence of stressors (Mittler, 2002; Lesser, 2006; Fijalkowski and Kwarciak-Kozlowska, 2020).

The increase in CAT activity in the controls of the strain *Pseudomonas* sp. CMA 6.9 throughout the evaluated growth phases (Figure 7A) was accompanied by the reduction of H_2_O_2_ (Figure 4), showing the efficiency of this enzyme in conditions without herbicides. The APX and GPX enzymes (Figures 7B,C) also showed a significant increase only in the mid-log stage, characterizing the cooperative action with CAT for the control of H_2_O_2_. The same efficiency in the control of H_2_O_2_ was not observed in treatments with the Boral^®^ herbicide (Figure 4). In B15 there were variations in the activities of three enzymes, which were not observed for B45, for which the activities were relatively the lowest of all (Figure 7). CAT is an important antioxidant enzyme in bacteria exposed to xenobiotics. The activity of this enzyme has been observed in freshwater bacterial biofilms exposed to the herbicide oxyfluorfen (Bonnineau et al., 2013), and *P. aeruginosa* PAO1 WT exposed to nicotine (Tang et al., 2018). Despite the importance of CAT, other enzymes participate in the metabolism of H_2_O_2_, such as glutathione peroxidases (Gebicka and Krych-Madej, 2019). Different *Chlamydomonas* strains showed variation at APX activities when exposed to methyl viologen, indicating that this enzyme contributes to tolerance to this herbicide (Tanaka et al., 2011). APX was also important in ERO control induced by heavy metals, such as zinc, in *Pseudomonas putida* (Hussein and Ho, 2013).

The joint action of CAT, APX, and GPX enzymes in controlling H_2_O_2_ in bacteria is described in only a few reports in the literature. These effects are limited to bacteria in modulating the activity of these enzymes in plants (Shahid and Khan, 2018). A strain of rhizosphere *Variovorax* sp. was considered tolerant to water stress because of its activity profile of antioxidative enzymes and used to stimulate these characteristics for a symbiotic plant. This strain showed high CAT activity and low APX during stress experiments (Kim et al., 2020). Even for CAT, one of the most studied enzymes in the control of oxidative stress, few reports have followed its activity in relation to the use of herbicides, such as an endophytic strain of *Klebsiella pneumoniae* isolated in evening primrose, used as a bio-herbicide (Kang et al., 2020). However, no similar reports were found on the modulation of the activity of these enzymes in the control of oxidative stress in *Pseudomonas* sp. CMA 6.9.

#### Enzymatic Activity of GST

Bacterial GST specializes in the detoxification of many molecules via conjugation by GSH (Allocati et al., 2009). Glutathione is an ROS reducing agent produced from cysteine and participates in the glutaredoxin system (Hufnagel et al., 2017). An important feature, however, has been studied in relation to GST, the regulation of cell signaling pathway lipid peroxidation products (Awasthi et al., 2017).

Glutathione S-transferase activity in *Pseudomonas* sp. CMA 6.9 (Figure 8) showed a relationship with MDA (Figure 5), which is little explored in the literature. Especially in H15 treatment, GST activity was proportional in the early-log and mid-log phases and inversely proportional in the early mid-log phase. GST may be related to the control of lipid peroxidation in this strain, in response to the stress induced mainly by the herbicide Heat^®^. Temperature stress can change the fluidity of membranes, which may be compensated by the action of desaturases, responsible for substitutions of saturated and unsaturated fatty acids, and GST may be related to the removal of lipids damaged by peroxide and restoration of the fluidity of the membranes (Kammerscheit et al., 2019).

Changes in the composition of membrane lipids were correlated with lower rates of MDA and changes in permeability in response to the toxicity of the herbicide mesotrione in *E. coli* (Olchanheski et al., 2014) and *B. megaterium* (Dobrzanski et al., 2018). The response system for this same herbicide in *P. ananatis* probably involved the formation of GST-mesotrione conjugates, altering lipid saturation and providing a protective effect for the bacterium by decreasing the permeability of membranes to the herbicide (Prione et al., 2016).

### Metabolic Plasticity as a Response System for *Pseudomonas* sp. CMA 6.9 to Boral^®^ and Heat^®^ Herbicides

#### Antioxidative Enzymes and H_2_O_2_

In the early-log phase of growth, the enzymes CAT, APX, and GPX had a direct proportional correlation (most obviously with APX) in the H45 treatment but was inversely proportional with B45. A positive correlation of enzymes activities with H_2_O_2_ was observed for B15 treatment, but inversely proportional for H15. These enzymes seem to work cooperatively by the control of peroxide, mainly in the treatment with Heat^®^.

The mid-log phase is characterized by a modulation like that of the initial growth phase in *Pseudomonas* sp. CMA 6.9, featuring more efficient control of these three enzymes over the stress generated by Heat^®^ than by Boral^®^, as already discussed in Section “Quantification of H_2_O_2_,” in terms of the toxicity of Boral^®^.

There are few reports on the activities of APX and GPX enzymes in microorganisms. In floating aquatic plants, *Hydrocharis dubia*, when exposed to different concentrations of glyphosate, the increase in H_2_O_2_ production was accompanied by an increase in CAT, APX, and GPX activities (Zhong et al., 2018).

#### Antioxidative Enzymes and Lipid Peroxidation

In this work we studied a little-explored relationship in the literature, the control of lipid peroxidation by the enzyme GST in bacteria (Awasthi et al., 2017). In the initial growth phase of *Pseudomonas* sp. CMA 6.9, there is a positive correlation between GST and MDA in treatment H15 but inversely proportional to treatment B15. In the early mid-log phase, GST activity is low for H45, while MDA is inversely proportional in treatments with Boral^®^. In the mid-log phase, as well as for the enzymes discussed in the previous phase, the pattern of interrelations resembles the early-log phase again. Therefore, it appears that lipid peroxidation in treatments with Boral^®^ is affected more by the activity of GST, although the amounts of H_2_O_2_ are higher, explaining the tolerance to this herbicide, as shown by the data on enzymatic kinetics (Figure 2) and viability (Figure 3). As noted, GST has the function of controlling oxidative stress induced by cytotoxic substances through conjugation with lipid aldehydes (Singhal et al., 2015).

#### Metabolic Plasticity

The biofilm strain *Pseudomonas* sp. CMA 6.9 is tolerant to the herbicide Heat^®^, based on the joint action of the enzymes CAT, APX, and GPX in the control of H_2_O_2_. The literature points out that previous exposure to herbicides can alter the sensitivity, diversity, and functioning of microorganism species in aquatic biofilms, such as in exposure to the herbicides promethrin (Polst et al., 2018) or alachlor (Paule et al., 2015). However, the strain studied in this work tolerated the toxicity and oxidative stress induced by Boral^®^ by means of another enzymatic system, GST, to control the amounts of MDA, even when this herbicide did not exert selective pressure at the isolation site. The genus *Pseudomonas* is metabolically versatile and plastic, with the ability to metabolize different toxic substances (Orellana-Saez et al., 2019). Thus, the two enzymatic systems of *Pseudomonas* sp. CMA 6.9 to deal with oxidative stress, induced by two different herbicides – one without prior selection – are related to two groups of genes linked to different antioxidative systems, possibly allowing tolerance to both herbicides as more general NTSR mechanisms (Comont et al., 2020). GSTs have already been proposed as NTSR mechanisms (Jugulam and Shyam, 2019; Gaines et al., 2020). In this work, in addition to GST, we are proposing the CAT, APX, and GST systems as NTSR mechanisms in *Pseudomonas* sp. CMA 6.9.

#### Approaches to Unresolved Issues

*Pseudomonas* sp. CMA 6.9, selected from the collection of bacteria isolated from biofilms in tanks used to wash pesticide packaging, was evaluated for enzyme systems that possibly induce tolerance to Boral^®^ and Heat^®^ herbicides, as shown by growth and viability kinetics data. The H_2_O_2_ and MDA data indicated the toxicity of these herbicides, especially Boral^®^. Although these herbicides act to inhibit the enzyme protoporphyrinogen oxidase, two systems of antioxidative responses were characteristic for each of them: CAT, APX, and GPX in the control of H_2_O_2_ induced by Heat^®^, present at the isolation site; and GST acting to control MDA in treatments with Boral^®^, not present at the isolation site. This modulation of the activity by different enzymes dependent on previous selection characterizes a system of metabolic plasticity. However, 67.2% of the isolates studied in this work were sensitive to these herbicides, suggesting that changes in diversity in soil and water microbiota may occur in the presence of pesticides. Meanwhile, diversity molecular studies revealed that trace metal contamination in water environment have higher impacts on the functional profiles than on the structure of biofilm communities (Coclet et al., 2021). The authors consider that biofilms are less impacted than planktonic communities to metal contamination due to a protection offered by the resistant species to all members in the community, and a wide range of specific mechanism of protection. Taking this in consideration, studies involving metabolome and identification of quorum-sensing signaling molecules should be carried out to test hypotheses regarding the modulations of the enzymatic response systems for the herbicides Boral^®^ and Heat^®^ in *Pseudomonas* sp. CMA 6.9. Biofilms containing this strain could protect the herbicide-sensitive isolates, opening a possibility of using biofilms containing biodegrade communities of biofilms in recovery from contaminated areas.

## Data Availability Statement

The datasets presented in this study can be found in online repositories. The names of the repository/repositories and accession number(s) can be found in the article/Supplementary Material.

## Author Contributions

AR, CS, and PF: data curation, formal analysis, investigation, methodology, software, visualization, and writing original draft, review, and editing. GC and MS: methodology. EO: investigation, methodology, and visualization. SP: writing, review, and editing. RO: conceptualization, data curation, formal analysis, resources, supervision, validation, and writing original draft, review, and editing. MP: conceptualization, data curation, formal analysis, funding acquisition, project administration, resources, supervision, validation, and writing original draft, review, and editing. All authors contributed to the article and approved the submitted version.

## Conflict of Interest

The authors declare that the research was conducted in the absence of any commercial or financial relationships that could be construed as a potential conflict of interest.
