# Supplementary material for: Herbicides Tolerance in a Pseudomonas Strain Is Associated With Metabolic Plasticity of Antioxidative Enzymes Regardless of Selection
Source: Front Microbiol. 2021 Jun 22;12:673211. doi: 10.3389/fmicb.2021.673211 (PMC8258386; doi:10.3389/fmicb.2021.673211)
Supplement: Supplementary file 1 [file Table_1.DOCX]

Supplementary Material 1

# Supplementary Data

**Protein profile SDS-PAGE**

The analysis of SDS-PAGE gels profiles from *Pseudomonas* sp. CMA 6.9 extracts showed no significant differences among the bands, demonstrating no contamination and high extraction quality.

**Total protein analysis**


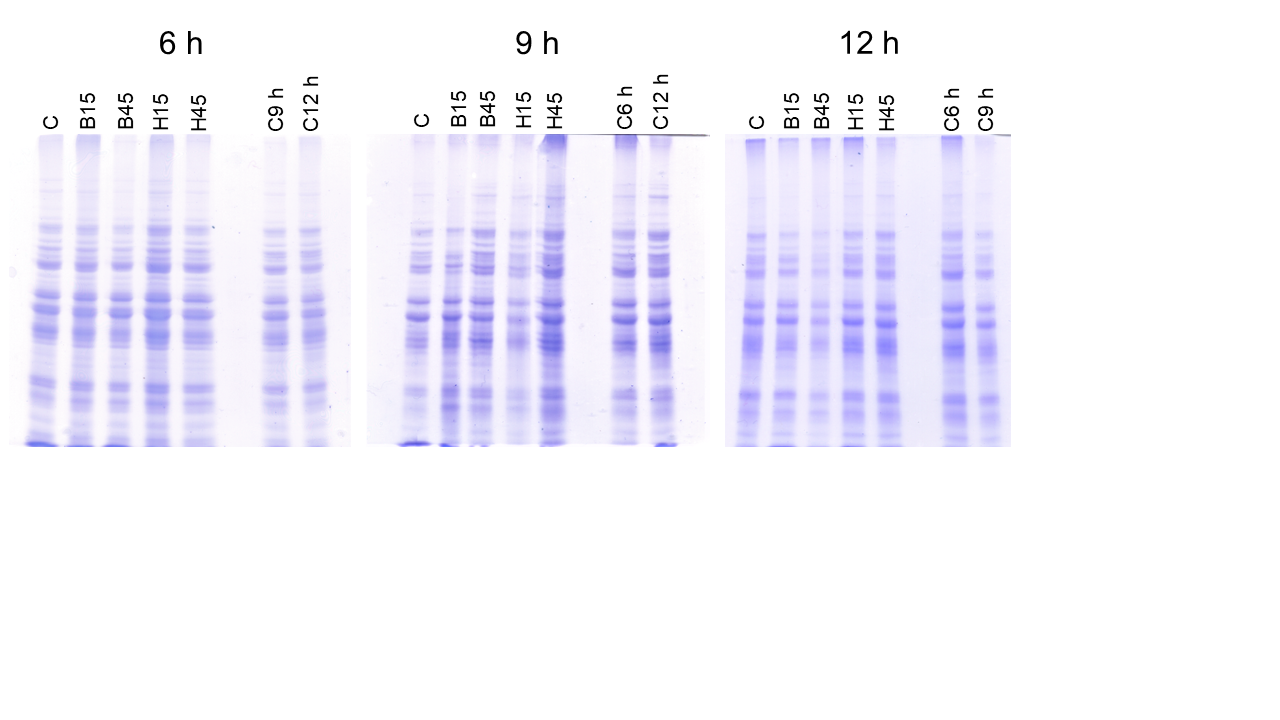


# Figure 1 - Analysis of total proteins in SDS-PAGE gel from *Pseudomonas* sp. CMA 6.9 in control (C) and treatments with the herbicides Heat (H15 and H45) and Boral (B15 and B45). Controls from the different times were inserted for bands intensity comparison, with C6 h = 6 hours control; C9 h = 9 hours control; C12 h = 12 hours control.
