# Supplementary material for: Herbicides Tolerance in a Pseudomonas Strain Is Associated With Metabolic Plasticity of Antioxidative Enzymes Regardless of Selection
Source: Front Microbiol. 2021 Jun 22;12:673211. doi: 10.3389/fmicb.2021.673211 (PMC8258386; doi:10.3389/fmicb.2021.673211)
Supplement: Supplementary file 2 [file Table_2.DOCX]

Supplementary Material 2

# Supplementary Data

## STATISTICAL ANALYSIS FOR GROWTH CURVE

> LDuncan (CURVE0h)

DUNCAN TEST TO COMPARE MEANS

Confidence Level: 0.95

Dependent Variable: zero

Variation Coefficient: 4.163336e-14 %

Independent Variable: Trat

Factors Means

0x 0.05 a

15xB 0.05 a

15xH 0.05 a

45xB 0.05 a

45xH 0.05 a

> LDuncan(CURVE1h)

DUNCAN TEST TO COMPARE MEANS

Confidence Level: 0.95

Dependent Variable: one

Variation Coefficient: 42.39962 %

Independent Variable: Trat

Factors Means

45xB 0.143333333333333 a

45xH 0.113333333333333 ab

15xH 0.0666666666666667 b

15xB 0.0633333333333333 b

0x 0.0566666666666667 b

> LDuncan(CURVE2h)

DUNCAN TEST TO COMPARE MEANS

Confidence Level: 0.95

Dependent Variable: two

Variation Coefficient: 21.12864 %

Independent Variable: Trat

Factors Means

45xB 0.226666666666667 a

45xH 0.16 b

0x 0.123333333333333 b

15xB 0.116666666666667 b

15xH 0.116666666666667 b

> LDuncan(CURVE3h)

DUNCAN TEST TO COMPARE MEANS

Confidence Level: 0.95

Dependent Variable: three

Variation Coefficient: 9.135831 %

Independent Variable: Trat

Factors Means

45xB 0.293333333333333 a

45xH 0.27 ab

15xH 0.23 bc

0x 0.226666666666667 bc

15xB 0.22 c

> LDuncan(CURVE4h)

DUNCAN TEST TO COMPARE MEANS

Confidence Level: 0.95

Dependent Variable: four

Variation Coefficient: 4.199189 %

Independent Variable: Trat

Factors Means

45xH 0.453333333333333 a

45xB 0.433333333333333 a

15xH 0.43 a

0x 0.42 ab

15xB 0.393333333333333 b

> LDuncan(CURVE5h)

DUNCAN TEST TO COMPARE MEANS

Confidence Level: 0.95

Dependent Variable: five

Variation Coefficient: 5.115309 %

Independent Variable: Trat

Factors Means

45xH 0.784333333333333 a

15xH 0.745666666666667 ab

0x 0.738666666666667 ab

15xB 0.73 ab

45xB 0.728 b

> LDuncan(CURVE6h)

DUNCAN TEST TO COMPARE MEANS

Confidence Level: 0.95

Dependent Variable: six

Variation Coefficient: 6.057448 %

Independent Variable: Trat

Factors Means

0x 1.38666666666667 a

15xB 1.37 ab

15xH 1.37 ab

45xB 1.35 ab

45xH 1.32666666666667 b

> LDuncan(CURVE7h)

DUNCAN TEST TO COMPARE MEANS

Confidence Level: 0.95

Dependent Variable: seven

Variation Coefficient: 7.059958 %

Independent Variable: Trat

Factors Means

15xH 1.77666666666667 a

0x 1.71333333333333 ab

45xB 1.69333333333333 ab

45xH 1.67666666666667 ab

15xB 1.63666666666667 b

> LDuncan(CURVE8h)

DUNCAN TEST TO COMPARE MEANS

Confidence Level: 0.95

Dependent Variable: eight

Variation Coefficient: 9.388831 %

Independent Variable: Trat

Factors Means

45xB 2.48 a

15xH 2.36666666666667 ab

0x 2.34666666666667 ab

15xB 2.29333333333333 ab

45xH 2.21 b

> LDuncan(CURVE9h)

DUNCAN TEST TO COMPARE MEANS

Confidence Level: 0.95

Dependent Variable: nine

Variation Coefficient: 9.04515 %

Independent Variable: Trat

Factors Means

45xB 3 a

15xH 2.73 ab

45xH 2.54333333333333 ab

0x 2.54 ab

15xB 2.53 b

> LDuncan(CURVE10h)

DUNCAN TEST TO COMPARE MEANS

Confidence Level: 0.95

Dependent Variable: ten

Variation Coefficient: 10.82591 %

Independent Variable: Trat

Factors Means

45xB 3.61 a

0x 3.20666666666667 ab

15xH 3.03666666666667 ab

15xB 2.94666666666667 ab

45xH 2.85 b

> LDuncan(CURVE11h)

DUNCAN TEST TO COMPARE MEANS

Confidence Level: 0.95

Dependent Variable: eleven

Variation Coefficient: 11.95877 %

Independent Variable: Trat

Factors Means

45xB 4.08 a

15xH 3.61666666666667 ab

0x 3.47 ab

15xB 3.38666666666667 ab

45xH 3.38 b

> LDuncan(CURVE12h)

DUNCAN TEST TO COMPARE MEANS

Confidence Level: 0.95

Dependent Variable: twelve

Variation Coefficient: 9.436362 %

Independent Variable: Trat

Factors Means

45xB 4.59 a

15xH 3.83333333333333 b

0x 3.79333333333333 b

15xB 3.79 b

45xH 3.75666666666667 b

> LDuncan(CURVE13h)

DUNCAN TEST TO COMPARE MEANS

Confidence Level: 0.95

Dependent Variable: thirteen

Variation Coefficient: 7.326331 %

Independent Variable: Trat

Factors Means

45xB 4.93 a

15xH 4.33 ab

0x 4.14 b b

45xH 4.09 b

15xB 4.07 b

> LDuncan(CURVE14h)

DUNCAN TEST TO COMPARE MEANS

Confidence Level: 0.95

Dependent Variable: fourteen

Variation Coefficient: 9.656846 %

Independent Variable: Trat

Factors Means

45xB 5.18333333333333 a

15xH 4.67 ab

15xB 4.33 ab

45xH 4.31333333333333 ab

0x 4.3 b

> LDuncan(CURVE15h)

DUNCAN TEST TO COMPARE MEANS

Confidence Level: 0.95

Dependent Variable: fiveteen

Variation Coefficient: 6.694895 %

Independent Variable: Trat

Factors Means

45xB 5.63 a

15xH 4.79 b

15xB 4.76333333333333 b

0x 4.64666666666667 b

45xH 4.64 b

> LDuncan(CURVE16h)

DUNCAN TEST TO COMPARE MEANS

Confidence Level: 0.95

Dependent Variable: sixteen

Variation Coefficient: 6.296074 %

Independent Variable: Trat

Factors Means

45xB 5.9 a

15xB 4.90333333333333 b

0x 4.89 b

15xH 4.79 b

45xH 4.71666666666667 b

> LDuncan(CURVE17h)

DUNCAN TEST TO COMPARE MEANS

Confidence Level: 0.95

Dependent Variable: seventeen

Variation Coefficient: 7.53836 %

Independent Variable: Trat

Factors Means

45xB 5.93333333333333 a

15xH 5.23666666666667 ab

0x 5.22666666666667 ab

15xB 5.21333333333333 ab

45xH 4.89 b

> LDuncan(CURVE18h)

DUNCAN TEST TO COMPARE MEANS

Confidence Level: 0.95

Dependent Variable: eighteen

Variation Coefficient: 6.829399 %

Independent Variable: Trat

Factors Means

45xB 6.30666666666667 a

15xH 5.39333333333333 b

15xB 5.20333333333333 b

45xH 5.08666666666667 b

0x 5.04 b

> LDuncan(CURVE19h)

DUNCAN TEST TO COMPARE MEANS

Confidence Level: 0.95

Dependent Variable: nineteen

Variation Coefficient: 6.971018 %

Independent Variable: Trat

Factors Means

45xB 6.40666666666667 a

15xH 5.47 b

45xH 5.19333333333333 b

15xB 5.15333333333333 b

0x 5.09333333333333 b

> LDuncan(CURVE20h)

DUNCAN TEST TO COMPARE MEANS

Confidence Level: 0.95

Dependent Variable: twenty

Variation Coefficient: 7.190676 %

Independent Variable: Trat

Factors Means

45xB 6.33666666666667 a

15xH 5.73333333333333 ab

15xB 5.40333333333333 b

0x 5.27333333333333 b

45xH 5.15333333333333 b

> LDuncan(CURVE21h)

DUNCAN TEST TO COMPARE MEANS

Confidence Level: 0.95

Dependent Variable: twenty one

Variation Coefficient: 4.116035 %

Independent Variable: Trat

Factors Means

45xB 6.62333333333333 a

15xH 6.46 a

45xH 6.14 ab

15xB 5.92 b

0x 5.9 b

> LDuncan(CURVE22h)

DUNCAN TEST TO COMPARE MEANS

Confidence Level: 0.95

Dependent Variable: twenty two

Variation Coefficient: 4.074496 %

Independent Variable: Trat

Factors Means

45xB 6.57333333333333 a

15xH 6.34333333333333 ab

0x 6.27666666666667 ab

15xB 6.24 ab

45xH 5.88333333333333 b

> LDuncan(CURVE23h)

DUNCAN TEST TO COMPARE MEANS

Confidence Level: 0.95

Dependent Variable: twenty three

Variation Coefficient: 4.396369 %

Independent Variable: Trat

Factors Means

15xH 7.04 a

45xB 6.885 ab

0x 6.59 abc

15xB 6.345 bc

45xH 6.25666666666667 c

> LDuncan(CURVE24h)

DUNCAN TEST TO COMPARE MEANS

Confidence Level: 0.95

Dependent Variable: twenty four

Variation Coefficient: 3.789813 %

Independent Variable: Trat

Factors Means

45xB 6.64 a

15xH 6.31 ab

0x 6.23666666666667 ab

45xH 6.17 ab

15xB 6.08666666666667 b

> LDuncan(CURVE25h)

DUNCAN TEST TO COMPARE MEANS

Confidence Level: 0.95

Dependent Variable: twenty five

Variation Coefficient: 3.800897 %

Independent Variable: Trat

Factors Means

45xB 6.77333333333333 a

0x 6.74333333333333 a

15xB 6.44333333333333 ab

45xH 6.22 b

15xH 6.10333333333333 b

> LDuncan(CURVE26h)

DUNCAN TEST TO COMPARE MEANS

Confidence Level: 0.95

Dependent Variable: twenty six

Variation Coefficient: 5.191849 %

Independent Variable: Trat

Factors Means

15xH 6.68333333333333 a

45xB 6.5 ab

45xH 6.34 ab

0x 6.32 ab

15xB 6.00333333333333 b

> LDuncan(CURVE27h)

DUNCAN TEST TO COMPARE MEANS

Confidence Level: 0.95

Dependent Variable: twenty seven

Variation Coefficient: 4.950535 %

Independent Variable: Trat

Factors Means

0x 6.60333333333333 a

45xB 6.49666666666667 ab

15xB 6.45 ab

45xH 6.41 ab

15xH 6.21 b

2.2 STATISTICAL ANALYSIS FOR VIABILITY

DUNCAN TEST TO COMPARE MEANS

Confidence Level: 0.95

Dependent Variable: Viability

Variation Coefficient: 26.43202 %

Independent Variable: Trat

Factors Means

45xH 6h 1.905e+10 a

0x 6h 12833333333.3333 ab

15xH 6h 12737500000 ab

45xB 6h 12379166666.6667 ab

15xB 6h 11366666666.6667 b

DUNCAN TEST TO COMPARE MEANS

Confidence Level: 0.95

Dependent Variable: Viability

Variation Coefficient: 12.28043 %

Independent Variable: Trat

Factors Means

0x 9h 23147222222.3333 a

45xB 9h 22470833333.3333 ab

15xH 9h 21688888889 ab

15xB 9h 20616666666.6667 ab

45xH 9h 19054166666.6667 b

DUNCAN TEST TO COMPARE MEANS

Confidence Level: 0.95

Dependent Variable: Viability

Variation Coefficient: 18.57002 %

Independent Variable: Trat

Factors Means

45xB 12h 27131250000 a

0x 12h 25765277777.6667 ab

15xH 12h 24939583333.5 ab

15xB 12h 24631250000 ab

45xH 1 2h 22062500000 b

DUNCAN TEST TO COMPARE MEANS

Confidence Level: 0.95

Dependent Variable: Viability

Variation Coefficient: 8.694769 %

Independent Variable: Trat

Factors Means

0x 12h 25765277777.6667 a

0x 9h 23147222222.3333 a

0x 6h 12833333333.3333 b

DUNCAN TEST TO COMPARE MEANS

Confidence Level: 0.95

Dependent Variable: Viability

Variation Coefficient: 25.37818 %

Independent Variable: Trat

Factors Means

15xB 12h 24631250000 a

15xB 9h 20616666666.6667 ab

15xB 6h 11366666666.6667 b

DUNCAN TEST TO COMPARE MEANS

Confidence Level: 0.95

Dependent Variable: Viability

Variation Coefficient: 20.45921 %

Independent Variable: Trat

Factors Means

45xB 12h 27131250000 a

45xB 9h 22470833333.3333 a

45xB 6h 12379166666.6667 b

DUNCAN TEST TO COMPARE MEANS

Confidence Level: 0.95

Dependent Variable: Viability

Variation Coefficient: 22.39326 %

Independent Variable: Trat

Factors Means

15xH 12h 24939583333.5 a

15xH 9h 21688888889 ab

15xH 6h 12737500000 b

DUNCAN TEST TO COMPARE MEANS

Confidence Level: 0.95

Dependent Variable: Viability

Variation Coefficient: 11.75656 %

Independent Variable: Trat

Factors Means

45xH 12h 22062500000 a

45xH 9h 19054166666.6667 ab

45xH 6h 1.905e+10 b

2.3 STATISTICAL ANALYSIS FOR h_2_o_2_

DUNCAN TEST TO COMPARE MEANS

Confidence Level: 0.95

Dependent Variable: H2O2

Variation Coefficient: 33.11082 %

Independent Variable: Trat

Factors Means

15xB 6h 2.98357 a

0x 6h 1.7222233335 b

15xH 6h 1.59123733333333 b

45xB 6h 1.440846 b

45xH 6h 1.20313066666667 b

DUNCAN TEST TO COMPARE MEANS

Confidence Level: 0.95

Dependent Variable: H2O2

Variation Coefficient: 11.7302 %

Independent Variable: Trat

Factors Means

15xB 9h 2.576058 a

45xB 9h 2.503288 a

15xH 9h 1.993898 ab

45xH 9h 1.833804 b

0x 9h 1.637325 b

DUNCAN TEST TO COMPARE MEANS

Confidence Level: 0.95

Dependent Variable: H2O2

Variation Coefficient: 16.66138 %

Independent Variable: Trat

Factors Means

15xB 12h 3.827702 a

45xB 12h 3.376528 a

45xH 12h 1.979344 b

15xH 12h 1.513616 b

0x 12h 1.2419413335 b

DUNCAN TEST TO COMPARE MEANS

Confidence Level: 0.95

Dependent Variable: H2O2

Variation Coefficient: 23.56051 %

Independent Variable: Trat

Factors Means

0x 6h 1.7222233335 a

0x 9h 1.637325 ab

0x 12h 1.2419413335 b

DUNCAN TEST TO COMPARE MEANS

Confidence Level: 0.95

Dependent Variable: H2O2

Variation Coefficient: 12.72641 %

Independent Variable: Trat

Factors Means

15xB 12h 3.827702 a

15xB 6h 2.98357 ab

15xB 9h 2.576058 b

DUNCAN TEST TO COMPARE MEANS

Confidence Level: 0.95

Dependent Variable: H2O2

Variation Coefficient: 10.72454 %

Independent Variable: Trat

Factors Means

45xB 12h 3.376528 a

45xB 9h 2.503288 b

45xB 6h 1.440846 c

DUNCAN TEST TO COMPARE MEANS

Confidence Level: 0.95

Dependent Variable: H2O2

Variation Coefficient: 17.17639 %

Independent Variable: Trat

Factors Means

15xH 9h 1.993898 a

15xH 12h 1.513616 ab

15xH 6h 1.193428 b

DUNCAN TEST TO COMPARE MEANS

Confidence Level: 0.95

Dependent Variable: H2O2

Variation Coefficient: 27.6005 %

Independent Variable: Trat

Factors Means

45xH 12h 1.979344 a

45xH 9h 1.833804 ab 45xH 6h 1.469954 b

## STATISTICAL ANALYSIS FOR MDA

DUNCAN TEST TO COMPARE MEANS

Confidence Level: 0.95

Dependent Variable: MDA

Variation Coefficient: 19.76456 %

Independent Variable: Trat

Factors Means

0x 6h 4.65 a

45xB 6h 4.2625 ab

15xH 6h 4.14625 ab

45xH 6h 2.59625 bc

15xB 6h 2.13125 c

DUNCAN TEST TO COMPARE MEANS

Confidence Level: 0.95

Dependent Variable: MDA

Variation Coefficient: 5.541365 %

Independent Variable: Trat

Factors Means

45xH 9h 5.38625 a

0x 9h 4.2625 b

15xH 9h 4.14625 b

15xB 9h 3.255 c

45xB 9h 2.48 d

DUNCAN TEST TO COMPARE MEANS

Confidence Level: 0.95

Dependent Variable: MDA

Variation Coefficient: 12.40041 %

Independent Variable: Trat

Factors Means

15xH 12h 4.495 a

0x 12h 3.60375 ab

15xB 12h 3.0225 b

45xH 12h 2.98375 b

45xB 12h 2.51875 b

DUNCAN TEST TO COMPARE MEANS

Confidence Level: 0.95

Dependent Variable: MDA

Variation Coefficient: 8.202874 %

Independent Variable: Trat

Factors Means

0x 6h 4.65 a

0x 9h 4.2625 ab

0x 12h 3.60375 b

DUNCAN TEST TO COMPARE MEANS

Confidence Level: 0.95

Dependent Variable: MDA

Variation Coefficient: 13.96245 %

Independent Variable: Trat

Factors Means

15xB 9h 3.255 a

15xB 12h 3.0225 ab

15xB 6h 2.13125 b

DUNCAN TEST TO COMPARE MEANS

Confidence Level: 0.95

Dependent Variable: MDA

Variation Coefficient: 21.12867 %

Independent Variable: Trat

Factors Means

45xB 6h 4.2625 a

45xB 12h 2.51875 ab

45xB 9h 2.48 b

DUNCAN TEST TO COMPARE MEANS

Confidence Level: 0.95

Dependent Variable: MDA

Variation Coefficient: 7.196571 %

Independent Variable: Trat

Factors Means

15xH 12h 4.495 a

15xH 6h 4.14625 ab

15xH 9h 4.14625 b

DUNCAN TEST TO COMPARE MEANS

Confidence Level: 0.95

Dependent Variable: MDA

Variation Coefficient: 17.20235 %

Independent Variable: Trat

Factors Means

45xH 9h 5.38625 a

45xH 12h 2.98375 b

45xH 6h 2.59625 b

## STATISTICAL ANALYSIS FOR CAT

DUNCAN TEST TO COMPARE MEANS

Confidence Level: 0.95

Dependent Variable: CAT

Variation Coefficient: 17.02226 %

Independent Variable: Trat

Factors Means

45xH 6h 1194.398405 a

0x 6h 1004.61125565 a

15xH 6h 986.20003685 a

15xB 6h 792.8940511 ab

45xB 6h 446.98415085 b

DUNCAN TEST TO COMPARE MEANS

Confidence Level: 0.95

Dependent Variable: CAT

Variation Coefficient: 17.45631 %

Independent Variable: Trat

Factors Means

0x 9h 1343.3714555 a

15xB 9h 1059.1858726 a

15xH 9h 574.4393602 b

45xB 9h 546.61847545 b

45xH 9h 446.71555065 b

DUNCAN TEST TO COMPARE MEANS

Confidence Level: 0.95

Dependent Variable: CAT

Variation Coefficient: 6.906182 %

Independent Variable: Trat

Factors Means

15xB 12h 1663.731251 a

0x 12h 1533.746596 a

45xH 12h 1190.7013275 b

15xH 12h 990.36519695 b

45xB 12h 447.27876635 c

DUNCAN TEST TO COMPARE MEANS

Confidence Level: 0.95

Dependent Variable: CAT

Variation Coefficient: 13.48701 %

Independent Variable: Trat

Factors Means

0x 12h 1533.746596 a

0x 9h 1343.3714555 ab

0x 6h 1004.61125565 b

DUNCAN TEST TO COMPARE MEANS

Confidence Level: 0.95

Dependent Variable: CAT

Variation Coefficient: 8.929781 %

Independent Variable: Trat

Factors Means

15xB 12h 1663.731251 a

15xB 9h 1059.1858726 b

15xB 6h 792.8940511 b

DUNCAN TEST TO COMPARE MEANS

Confidence Level: 0.95

Dependent Variable: CAT

Variation Coefficient: 27.50045 %

Independent Variable: Trat

Factors Means

45xB 9h 546.61847545 a

45xB 12h 447.27876635 ab

45xB 6h 446.98415085 b

DUNCAN TEST TO COMPARE MEANS

Confidence Level: 0.95

Dependent Variable: CAT

Variation Coefficient: 13.37494 %

Independent Variable: Trat

Factors Means

15xH 12h 990.36519695 a

15xH 6h 986.20003685 a

15xH 9h 574.4393602 b

DUNCAN TEST TO COMPARE MEANS

Confidence Level: 0.95

Dependent Variable: CAT

Variation Coefficient: 9.968747 %

Independent Variable: Trat

Factors Means

45xH 6h 1194.398405 a

45xH 12h 1190.7013275 a

45xH 9h 446.71555065 b

## STATISTICAL ANALYSIS FOR APX

DUNCAN TEST TO COMPARE MEANS

Confidence Level: 0.95

Dependent Variable: APX

Variation Coefficient: 11.73582 %

Independent Variable: Trat

Factors Means

45xH 6h 325.87389685 a

15xH 6h 282.2199892 ab

0x 6h 225.26497895 bc

15xB 6h 186.1861244 c

45xB 6h 175.9459965 c

DUNCAN TEST TO COMPARE MEANS

Confidence Level: 0.95

Dependent Variable: APX

Variation Coefficient: 8.062393 %

Independent Variable: Trat

Factors Means

15xB 9h 342.6732688 a

15xH 9h 321.1015971 a

45xH 9h 291.84173215 a

0x 9h 215.1559393 b

45xB 9h 198.7398032 b

DUNCAN TEST TO COMPARE MEANS

Confidence Level: 0.95

Dependent Variable: APX

Variation Coefficient: 14.2454 %

Independent Variable: Trat

Factors Means

15xB 12h 427.97872495 a

0x 12h 341.38284895 ab

15xH 12h 259.68600825 bc

45xH 12h 209.8512673 c

45xB 12h 171.11281065 c

DUNCAN TEST TO COMPARE MEANS

Confidence Level: 0.95

Dependent Variable: APX

Variation Coefficient: 15.04398 %

Independent Variable: Trat

Factors Means

0x 12h 341.38284895 a

0x 6h 225.26497895 ab

0x 9h 215.1559393 b

DUNCAN TEST TO COMPARE MEANS

Confidence Level: 0.95

Dependent Variable: APX

Variation Coefficient: 11.84992 %

Independent Variable: Trat

Factors Means

15xB 12h 427.97872495 a

15xB 9h 342.6732688 a

15xB 6h 186.1861244 b

DUNCAN TEST TO COMPARE MEANS

Confidence Level: 0.95

Dependent Variable: APX

Variation Coefficient: 11.08168 %

Independent Variable: Trat

Factors Means

45xB 9h 198.7398032 a

45xB 6h 175.9459965 ab

45xB 12h 171.11281065 b

DUNCAN TEST TO COMPARE MEANS

Confidence Level: 0.95

Dependent Variable: APX

Variation Coefficient: 7.181011 %

Independent Variable: Trat

Factors Means

15xH 9h 321.1015971 a

15xH 6h 282.2199892 ab

15xH 12h 259.68600825 b

DUNCAN TEST TO COMPARE MEANS

Confidence Level: 0.95

Dependent Variable: APX

Variation Coefficient: 11.55532 %

Independent Variable: Trat

Factors Means

45xH 6h 325.87389685 a

45xH 9h 291.84173215 ab

45xH 12h 209.8512673 b

## STATISTICAL ANALYSIS FOR GPX

DUNCAN TEST TO COMPARE MEANS

Confidence Level: 0.95

Dependent Variable: GPX

Variation Coefficient: 11.86843 %

Independent Variable: Trat

Factors Means

45xH 6h 134.40286405 a

45xB 6h 103.120997435 b

0x 6h 102.696563275 b

15xB 6h 73.54986634 bc

15xH 6h 70.453403255 c

DUNCAN TEST TO COMPARE MEANS

Confidence Level: 0.95

Dependent Variable: GPX

Variation Coefficient: 24.98728 %

Independent Variable: Trat

Factors Means

15xB 9h 128.7238937 a

15xH 9h 66.957035915 b

0x 9h 61.677170245 b

45xB 9h 58.05765458 b

45xH 9h 46.67698072 b

DUNCAN TEST TO COMPARE MEANS

Confidence Level: 0.95

Dependent Variable: GPX

Variation Coefficient: 16.45191 %

Independent Variable: Trat

Factors Means

0x 12h 122.1906297 a

45xH 12h 103.735884565 ab

15xB 12h 92.27923383 ab

15xH 12h 78.62684709 bc

45xB 12h 46.76895698 c

DUNCAN TEST TO COMPARE MEANS

Confidence Level: 0.95

Dependent Variable: GPX

Variation Coefficient: 20.51713 %

Independent Variable: Trat

Factors Means

0x 12h 122.1906297 a

0x 6h 102.696563275 ab

0x 9h 61.677170245 b

DUNCAN TEST TO COMPARE MEANS

Confidence Level: 0.95

Dependent Variable: GPX

Variation Coefficient: 19.16865 %

Independent Variable: Trat

Factors Means

15xB 9h 128.7238937 a

15xB 12h 92.27923383 ab

15xB 6h 73.54986634 b

DUNCAN TEST TO COMPARE MEANS

Confidence Level: 0.95

Dependent Variable: GPX

Variation Coefficient: 12.31193 %

Independent Variable: Trat

Factors Means

45xB 6h 103.120997435 a

45xB 9h 58.05765458 b

45xB 12h 46.76895698 b

DUNCAN TEST TO COMPARE MEANS

Confidence Level: 0.95

Dependent Variable: GPX

Variation Coefficient: 12.02346 %

Independent Variable: Trat

Factors Means

15xH 12h 78.62684709 a

15xH 6h 70.453403255 ab

15xH 9h 66.957035915 b

DUNCAN TEST TO COMPARE MEANS

Confidence Level: 0.95

Dependent Variable: GPX

Variation Coefficient: 16.14356 %

Independent Variable: Trat

Factors Means

45xH 6h 134.40286405 a

45xH 12h 103.735884565 a

45xH 9h 46.67698072 b

## STATISTICAL ANALYSIS FOR GST

DUNCAN TEST TO COMPARE MEANS

Confidence Level: 0.95

Dependent Variable: GST

Variation Coefficient: 13.26515 %

Independent Variable: Trat

Factors Means

15xH 6h 0.0128333125 a

45xH 6h 0.0109976926666667 a

45xB 6h 0.0082739375 b

15xB 6h 0.0050734905 c

0x 6h 0.0042606755 c

DUNCAN TEST TO COMPARE MEANS

Confidence Level: 0.95

Dependent Variable: GST

Variation Coefficient: 12.46648 %

Independent Variable: Trat

Factors Means

0x 9h 0.0127960845 a

15xH 9h 0.0127408295 a

45xB 9h 0.0055070605 b

15xB 9h 0.0043163445 bc

45xH 9h 0.0019160045 cd

DUNCAN TEST TO COMPARE MEANS

Confidence Level: 0.95

Dependent Variable: GST

Variation Coefficient: 15.39607 %

Independent Variable: Trat

Factors Means

15xH 12h 0.006771323 a

0x 12h 0.00556853 a

15xB 12h 0.0035200225 b

45xH 12h 0.003011318 b

45xB 12h 0.0023232995 b

DUNCAN TEST TO COMPARE MEANS

Confidence Level: 0.95

Dependent Variable: GST

Variation Coefficient: 9.313809 %

Independent Variable: Trat

Factors Means

0x 9h 0.0127960845 a

0x 12h 0.00556853 b

0x 6h 0.0042606755 b

DUNCAN TEST TO COMPARE MEANS

Confidence Level: 0.95

Dependent Variable: GST

Variation Coefficient: 18.41192 %

Independent Variable: Trat

Factors Means

15xB 6h 0.0050734905 a

15xB 9h 0.0043163445 ab

15xB 12h 0.0035200225 b

DUNCAN TEST TO COMPARE MEANS

Confidence Level: 0.95

Dependent Variable: GST

Variation Coefficient: 21.37397 %

Independent Variable: Trat

Factors Means

45xB 6h 0.0082739375 a

45xB 9h 0.0055070605 ab

45xB 12h 0.0023232995 b

DUNCAN TEST TO COMPARE MEANS

Confidence Level: 0.95

Dependent Variable: GST

Variation Coefficient: 5.473908 %

Independent Variable: Trat

Factors Means

15xH 6h 0.0128333125 a

15xH 9h 0.0127408295 a

15xH 12h 0.006771323 b

DUNCAN TEST TO COMPARE MEANS

Confidence Level: 0.95

Dependent Variable: GST

Variation Coefficient: 19.63868 %

Independent Variable: Trat

Factors Means

45xH 6h 0.0109976926666667 a

45xH 12h 0.003011318 b

45xH 9h 0.0019160045 b

## GROUPING STATISTICS OF PCA ANALYSIS

Confidence Levels – Codes:

0 ‘***’ 0.001 ‘**’ 0.01 ‘*’ 0.05 ‘.’ 0.1 ‘ ’ 1

### - 6 h

PC1 PC2 r2 Pr(>r)

H2O2 0.43359 0.90111 0.9107 0.002 **

CAT -0.85797 0.51370 0.9581 0.001 ***

APX -0.64429 0.76478 0.8757 0.001 ***

GPX -0.99253 0.12200 0.8309 0.002 **

GST -0.70512 -0.70909 0.7975 0.002 **

### - 9 h

PC1 PC2 r2 Pr(>r)

H2O2 0.67248 -0.74012 0.5961 0.027 *

CAT 0.57530 0.81794 0.7851 0.002 **

APX 0.80189 -0.59747 0.4333 0.130

GPX 0.99938 -0.03526 0.9506 0.001 ***

GST 0.21221 0.97722 0.7209 0.008 **

### - 12 h

PC1 PC2 r2 Pr(>r)

H2O2 0.43359 0.90111 0.9107 0.002 **

CAT -0.85797 0.51370 0.9581 0.001 ***

APX -0.64429 0.76478 0.8757 0.002 **

GPX -0.99253 0.12200 0.8309 0.005 **

GST -0.70512 -0.70909 0.7975 0.003 **
