# Supplementary material for: Herbicides Tolerance in a Pseudomonas Strain Is Associated With Metabolic Plasticity of Antioxidative Enzymes Regardless of Selection
Source: Front Microbiol. 2021 Jun 22;12:673211. doi: 10.3389/fmicb.2021.673211 (PMC8258386; doi:10.3389/fmicb.2021.673211)
Supplement: Supplementary file 3 [file Table_3.DOCX]

Supplementary Material 3

# Supplementary Data

## Preparation of samples for degradation analysis

To determine the herbicide degradation capacity, *Pseudomonas* sp. CMA 6.9 was inoculated as already described (section 2.5.1). After 24 h, the cells were centrifuged at 8000 g for 5 min at 4 °C. The supernatant was discarded, and the concentrated cells were resuspended in 10 mL of LB. The 1x herbicide concentration was added to this sample, and the cells were incubated as already described. Samples of 1 mL were collected from the culture medium at 0 and 24 h of incubation and centrifuged at 12,000 g for 5 min. The supernatant was frozen at -80 °C. The samples were filter-sterilized before being injected in LC-MS/MS. The analyzes were performed on the Acquity and Xevo TQD model (Waters Corporation, Milford, MA, USA) equipped with binary pumps, a vacuum degasser, a SIL-HTc automatic sampler and column oven, using an ACQUITY UPLC® BEH C18 2.1 column x 50 mm (1.7 µm porosity), maintained at room temperature. The mass spectrometer was the QTRAP 4000 triple quadrupole linear ion traction, equipped with a TurboIon source, used in negative ion electrospray mode. The experiments were carried out in triplicates, comparing the samples at 0 and 24 h, to observe the decrease in the amount of the herbicide after incubation time.

## Degradation analysis in LC-MS/MS

The liquid chromatography analyzes coupled to mass spectrometry (LC-MS/MS) were performed with the injection of 10 µL of the extracts prepared as described in the previous section, in the LC-MS/MS Acquity and Xevo TQD Waters equipment, at a flow rate 0.2 mL min^-1^. The UPLC system consisted of 0.1% formic acid (mobile phase A) in ultra-pure water, and a mobile phase B consisting of acetonitrile standard HPLC. The gradient profile was as follows: isocratic over 1 min, a linear gradient from 10% to 90% B over 4 min, followed by 100% B over 1 min, with a flow rate of 0.2 mL/min^-1^. The column was rebalanced for 2 min. The instrument was operated in the ESI negative mode, and data was acquired in the multiple reaction monitoring (MRM) mode. The conditions for the saflufenacil active molecule were as follows: the capillary voltage was fixed at 2.9 kV and the cone voltage 55 V; the source temperature and the desolvation temperature were maintained at 150 °C and 350 °C, respectively; the desolvation gas was adjusted to a flow of 650 L h^−1^; 499.2 (m/z) was selected as the precursor ion, and its quantitative and qualitative ions were 348 (m/z) and 328 (m/z), respectively; the collision energies were 32 V and 41 V, respectively. For UPLC analysis, Masslynx NT v.4.1 (Waters) software was used to process data.

The conditions for the sulfentrazone active molecule were: the capillary voltage was fixed at 2.7 kV and the cone voltage 55 V; the source temperature and the desolvation temperature were maintained at 150 °C and 350 °C, respectively; the desolvation gas was adjusted to a flow of 650 L h^−1^; 385 (m/z) was selected as the precursor ion, and its quantitative and qualitative ions were 307 (m/z) and 199.1 (m/z), respectively; the collision energies were 25 V and 40 V, respectively. For UPLC analysis, Masslynx NT v.4.1 (Waters) software was used to process data.
